# A scoping review of theories, models and frameworks used or proposed to evaluate knowledge mobilization strategies

**DOI:** 10.1186/s12961-023-01090-7

**Published:** 2024-01-10

**Authors:** Saliha Ziam, Sèverine Lanoue, Esther McSween-Cadieux, Mathieu-Joël Gervais, Julie Lane, Dina Gaid, Laura Justine Chouinard, Christian Dagenais, Valéry Ridde, Emmanuelle Jean, France Charles Fleury, Quan Nha Hong, Ollivier Prigent

**Affiliations:** 1 School of Business Administration, Université TÉLUQ, Montreal, Canada; 2 Department of School and Social Adaptation Studies, Faculty of Education, Université de Sherbrooke, Sherbrooke, Canada; 3 Department of Psychology, Université du Québec à Montréal, Montreal, Canada; 4 Centre RBC d’expertise Universitaire en Santé Mentale, Université de Sherbrooke, Sherbrooke, Canada; 5 School of Rehabilitation, Faculty of Medicine, Université de Montréal, Montreal, Canada; 6 Department of Psychology, Université de Montréal, Montreal, Canada; 7 Université Paris Cité, IRD (Institute for Research on Sustainable Development, CEPED, Paris, France; 8 Institute of Health and Development (ISED), Cheikh Anta Diop University, Dakar, Senegal; 9 Public Health Intelligence and Knowledge Translation Division, Public Health Agency of Canada, Ottawa, Canada; 10 Coordinator of the Interregional Consortium of Knowledge in Health and Social Services (InterS4), Rimouski, Canada

**Keywords:** Knowledge mobilization, Knowledge translation, Theories, models, and frameworks, Evaluation, Scoping review

## Abstract

**Background:**

Evaluating knowledge mobilization strategies (KMb) presents challenges for organizations seeking to understand their impact to improve KMb effectiveness. Moreover, the large number of theories, models, and frameworks (TMFs) available can be confusing for users. Therefore, the purpose of this scoping review was to identify and describe the characteristics of TMFs that have been used or proposed in the literature to evaluate KMb strategies.

**Methods:**

A scoping review methodology was used. Articles were identified through searches in electronic databases, previous reviews and reference lists of included articles. Titles, abstracts and full texts were screened in duplicate. Data were charted using a piloted data charting form. Data extracted included study characteristics, KMb characteristics, and TMFs used or proposed for KMb evaluation. An adapted version of Nilsen (Implement Sci 10:53, 2015) taxonomy and the Expert Recommendations for Implementing Change (ERIC) taxonomy (Powell et al. in Implement Sci 10:21, 2015) guided data synthesis.

**Results:**

Of the 4763 search results, 505 were retrieved, and 88 articles were eligible for review. These consisted of 40 theoretical articles (45.5%), 44 empirical studies (50.0%) and four protocols (4.5%). The majority were published after 2010 (*n* = 70, 79.5%) and were health related (*n* = 71, 80.7%). Half of the studied KMb strategies were implemented in only four countries: Canada, Australia, the United States and the United Kingdom (*n* = 42, 47.7%). One-third used existing TMFs (*n* = 28, 31.8%). According to the adapted Nilsen taxonomy, process models (*n* = 34, 38.6%) and evaluation frameworks (*n* = 28, 31.8%) were the two most frequent types of TMFs used or proposed to evaluate KMb. According to the ERIC taxonomy, activities to “train and educate stakeholders” (*n* = 46, 52.3%) were the most common, followed by activities to “develop stakeholder interrelationships” (*n* = 23, 26.1%). Analysis of the TMFs identified revealed relevant factors of interest for the evaluation of KMb strategies, classified into four dimensions: context, process, effects and impacts.

**Conclusions:**

This scoping review provides an overview of the many KMb TMFs used or proposed. The results provide insight into potential dimensions and components to be considered when assessing KMb strategies.

**Supplementary Information:**

The online version contains supplementary material available at 10.1186/s12961-023-01090-7.

## Contribution to the literature

- The evaluation of KMb strategies is a critical dimension of the KMb process that is still poorly documented and warrants researchers’ attention.
- Our review identified the most common theories, models and frameworks (TMFs) proposed or used to assess KMb strategies and the main components to consider when evaluating a KMb strategy.
- By developing an integrative reference framework, this work contributes to improving organizations’ capacity to evaluate their KMb initiatives.

## Background

It is widely recognized that research evidence has the potential to inform, guide, and improve practices, decisions, and policies [1]. Unfortunately, for diverse reasons, the best available evidence is still too seldom taken into account and used [2–7]. The field of research on knowledge mobilization (KMb) has been growing rapidly since the early 2000s [2, 3, 8–11]. Its purpose is to better understand how to effectively promote and support evidence use.

Knowledge mobilization is one of many terms and concepts developed over recent decades to describe processes, strategies, and actions to bridge the gap between research and practice. Other common terms often paired interchangeably with the term “knowledge” are “translation”, “transfer”, “exchange”, “sharing” and “dissemination”, among others. [12, 13]. Some are more closely linked than others to specific fields or jurisdictions. For this study, we adopted the term knowledge mobilization (KMb) because it conveys the notions of complexity and multidirectional exchanges that characterize research-to-action processes. We used it as an umbrella concept that encompasses the efforts made to translate knowledge into concrete actions and beneficial impacts on populations [1]. Moreover, the term KMb is also used by research funding agencies in Canada to emphasize the medium- and long-term effects that research knowledge or research results can have on potential users [1, 14].

KMb represents all processes from knowledge creation to action and includes all strategies implemented to facilitate these processes [14]. A KMb strategy is understood as a coordinated set of activities to support evidence use, such as dissemination activities to reach target audiences (for example, educational materials, practical guides, decision support tools) or activities to facilitate knowledge application in a specific context and support professional behaviour change (for example, community of practice, educational meetings, audits and feedback, reminders, deliberative dialogues) [15]. A KMb process may vary in intensity, complexity or actor engagement depending on the nature of the research knowledge and the needs and preferences of evidence users [7].

KMb is considered a complex process, in that numerous factors can facilitate or hinder its implementation and subsequent evidence use. The past two decades have seen the emergence of a deeper understanding of these factors [2, 3, 16]. These may be related to the knowledge mobilized (for example, relevance, reliability, clarity, costs), the individuals involved in the KMb process (for example, openness to change, values, time available, resources), the KMB strategies (for example, fit with stakeholder needs and preferences, regular interactions, trust relationships, timing), and organizational and political contexts (for example, culture of evidence use, leadership, resources) [2, 6, 17, 18]. However, more studies are needed to understand which factors are more important in which contexts, and to evaluate the effects of KMb strategies.

On this last point, while essential, it is often very complex to study KMb impacts empirically to demonstrate the effectiveness of KMb strategies [19–21]. Partly for this reason, high-quality studies that evaluate process, mechanisms and effects of KMb strategies are still relatively rare [2, 22–25]. As a result, knowledge about the effectiveness of different KMb strategies remains limited [10, 17, 19, 23, 26–28] and their development cannot be totally evidence informed [3, 19, 20, 23, 29, 30], which may seem incompatible with the core values and principles of KMb.

The growing interest in KMb has led to an impressive proliferation of conceptual propositions, such as theories, models and frameworks (TMF) [2, 3, 9, 11, 12, 31, 32]. Many deplore the fact that these are poorly used [11, 30, 33] and insist on the need to test, refine and integrate existing ones [3, 31, 34]. Indeed, the conceptual and theoretical development of the field has outpaced its empirical development. This proliferation appears to have created confusion among certain users, such as organizations that need to evaluate their KMb strategies. Besides implementing and funding KMb strategies, knowledge organizations such as granting agencies, governments and public organizations, universities and health authorities are often required to demonstrate the impact of their strategies [21, 35, 36]. Yet this can be a significant challenge [20, 23, 29]. They may have difficulty knowing which TMFs to choose, in what context and how to use them effectively in their evaluation process [12, 37].

Indeed, the evaluation of KMb strategies is still relatively poorly documented, with respect to the phases of their development and implementation. Our aim in this scoping review is to clarify, conceptually and methodologically, this crucial dimension of the KMb process. This would help organizations gain access to evidence-based, operational and easy-to-use evaluation toolkits for assessing the impacts of their KMb strategies.

## Objectives

To survey the available knowledge on evaluation practices for KMb strategies, we conducted a scoping review. According to Munn et al. [38], a scoping review is indicated to identify the types of available evidence and knowledge gaps, to clarify concepts in the literature and to identify key characteristics or factors related to a concept. This review methodology also allows for the inclusion of a diversity of publications, regardless of their nature or research design, to produce the most comprehensive evidence mapping possible [39]. The objective of the scoping review was to identify and describe the characteristics of theories, models and frameworks (TMFs) used or proposed to evaluate KMb strategies. The specific research questions were:

1. What TMFs to evaluate KMb strategies exist in the literature?
2. What KMb strategies do they evaluate (that is types of KMb objectives, activities, target audiences)?
3. What dimensions and components are included in these TMFs?

## Methods

This scoping review was conducted based on the five steps outlined by Arksey and O’Malley [39]: (1) formulating the research questions; (2) identifying relevant studies; (3) selecting relevant studies; (4) extracting and charting data; and (5) analysing, collating, summarizing and presenting the data. Throughout the process, researchers and knowledge users (KMb practitioners) were involved in decisions regarding the research question, search strategy, selection criteria for studies and categories for data charting. We followed the Preferred Reporting Items for Systematic reviews and Meta-Analyses extension for Scoping Reviews (PRISMA-ScR) guidelines [40]. No protocol was registered for this review.

### Search strategy and information sources

The search strategy was developed, piloted and refined in consultation with our team’s librarian. Search terms included controlled vocabulary and keywords related to three main concepts: (1) knowledge mobilization (for example [knowledge or evidence or research] and transfer, translation, diffusion, dissemination, mobilization, implementation science, exchange, sharing, use, uptake, evidence-based practice, research-based evidence), (2) evaluation (for example, evaluat*, measur*, impact, outcome, assess, apprais*, indicator) and (3) TMF (for example, framework*, model*, method*, guide*, theor*). See Additional file 1 for the search terms and strategies used in the electronic searches.

The following databases were searched from January 2000 to August 2023: MEDLINE (Ovid), PsycInfo (Ovid), ERIC (ProQuest), Sociological Abstracts (ProQuest), Dissertations & Theses (Proquest), Érudit and Cairn. These databases were chosen to identify relevant references in the health, education and social fields. Several search strategies were tested by the librarian to optimize the retrieval of citations known to the investigators and to increase the likelihood that all relevant studies would be retrieved. We also searched reference lists of included articles and previous systematic reviews [11, 12, 15, 41].

### Eligibility criteria

A publication was considered eligible if it (1) presented or used a theory, model, or framework (TMF), (2) described dimensions or specific components to consider in the evaluation of KMb strategies, (3) presented or discussed KMb strategies or activities (any initiatives to improve evidence use), and (4) proposed outcomes that might result directly or indirectly from the KMb strategies. Studies were excluded from analysis if they (1) presented a TMF to assess the impact of research without mentioning KMb strategies or an intervention not related to KMb and (2) presented evaluation dimensions or components that could not be generalized. We considered publications in English or French. All types of articles and study designs were eligible, including study protocols.

### Study selection

The results of the literature search were imported into Covidence, which the review team used for screening. After duplicate articles were removed, the titles and abstracts were screened independently by two of the three reviewers (EMC, MJG, GL). Publications identified as potentially relevant were retrieved in full text and screened independently by three reviewers (EMC, MJG, GL). Discrepancies regarding the inclusion of any publication were resolved through discussion and consensus among reviewers. The principal investigator (SZ) validated the final selection of articles.

### Data synthesis

A data charting form was developed in Microsoft Excel and piloted by the research team. Data extracted included study characteristics (authors, authors’ country of affiliation, year, journal, discipline, article type, study setting, study aim), KMb strategies of interest, KMb objectives, KMb target audiences and TMFs used or proposed for KMb evaluation (existing or new TMF, specific dimensions or components of TMF and so on). Data were extracted by a single reviewer (SL, JC or OP) and validated by a second reviewer (SZ). Disagreements were discussed between reviewers and resolved by consensus. No quality appraisal of included studies was conducted, as this is optional in scoping reviews and the purpose was only to describe the content of identified TMFs [42].

### Data analysis and presentation of results

Data were summarized according to study characteristics, KMb strategy characteristics (activities, objectives, target audiences), types of TMFs, and dimensions or components to consider for KMb evaluation. Disagreements during the process were discussed and resolved through consensus (SL, DG, SZ). A KMb strategy might have one or more objectives and include one or more activities. Thus, the objectives and activities of the KMb strategies extracted from the selected studies were summarized based on existing categorizations. The categorization of KMb objectives was inspired by Gervais et al. [15] and Farkas et al. [43] (Table 1).

**Table 1:** Categories of KMb strategy objectives

Category	Description
Expose – inform	Exposure and dissemination of knowledge
Change attitude	Enable experience, raise awareness, change beliefs
Inform and influence decision-making	Support decision-makers, increase knowledge use in decision-making
Improve practices	Increase expertise, competence and use of knowledge
Foster collaboration	Build partnerships and improve communication and exchange

Adapted from Gervais et al. [15] and Farkas et al. [43]

The KMb activities were categorized according to the Expert Recommendations for Implementing Change (ERIC) taxonomy [44]. The activities were first classified according to the full taxonomy and then grouped into the nine categories proposed by Waltz et al. [45] (Table 2).

**Table 2:** Categories used to classify KMb activities

Category	Description
Train and educate stakeholders	Conduct ongoing training, develop educational materials
Develop stakeholder interrelationships	Conduct consensus discussion, develop academic partnership
Use evaluative and iterative strategies	Audit and provide feedback, conduct needs assessment
Provide interactive assistance	Facilitate, provide clinical supervision
Adapt and tailor to context	Tailor strategies, promote adaptability
Engage consumers	Involve patient and family members, use mass media
Change infrastructure	Change service sites, mandate change
Utilize financial strategies	Develop disincentives, alter allowance structure
Support clinicians	Create new clinical teams, develop resource-sharing agreements

Waltz et al. [45]; Powell et al. [44]

The TMFs were categorized according to the categories of theoretical approaches described by Nilsen [32]: process models, evaluation frameworks, determinant frameworks and classic theories (Table 3). The category “implementation theories” originally described by Nilsen [32] was not used because we did not identify any article that fit this category. We also added a category named “logic models” due to the nature of the identified TMFs. Logic models are often used in theory-driven evaluation approaches and are usually developed to show the links among inputs (resources), activities and outputs (outcomes and short-, medium- and long-term effects) [46].

**Table 3:** Categories used to classify the identified TMFs

Category	Description and examples
Process models	Describe steps or stages to translate evidence into action and offer practical guidance for KMb planning and execution (for example, the “Knowledge-to-Action framework” by Graham et al. [9])
Evaluation frameworks	Describe elements or components that could be evaluated to determine KMb implementation success (for example, “RE-AIM” by Glasgow et al. [47])
Determinant frameworks	Specify determinants that can act as barriers and enablers to understand or explain what influences KMb implementation and outcomes (for example, “PARISH” by Kitson et al. [48])
Classic theories	Theories that originate from other fields (for example, psychology sociology) and can be applied to understanding KMb processes (for example, social cognitive theories)
Logic models	Graphical representation of the links between inputs, activities, outputs and expected outcomes of a specific KMb strategy (for example, “CNODES knowledge translation logic model” by Sketris et al. [49])

Adapted from Nilsen [32]

Finally, the content extracted from the TMFs was analysed using mainly an inductive method. This method allows, among other things, to develop a reference framework or a model from the emerging categories that are evident in the text data [50].

The classification of concepts is the result of multiple readings and interpretations. The concepts associated with each dimension of the framework were classified according to their meaning. Similar concepts were grouped together to form components. These grouped components were then associated with the subdimensions and main dimensions of the framework.

## Results

### Search results

**Fig. 1:**
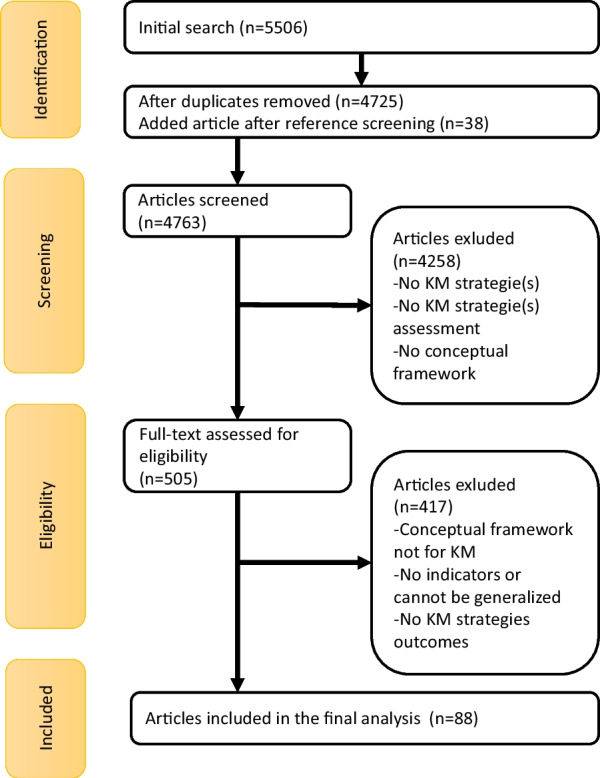
PRISMA flowchart summarizing search strategy and selection results [40]

The searches yielded 4763 articles. Of those, 4258 were excluded during the title and abstract screening. Of the 505 full-text articles, we retained 88 in our final sample. The results of the search and selection processes (PRISMA flowchart) are summarized in Fig. 1.

### Publication characteristics

Most articles were published after 2010 (*n* = 70, 79.5%), with an average of 5 articles per year between 2010 and 2023 compared with an average of 2.1 articles per year between 2001 and 2009; there were no eligible articles from 2000. The search was conducted in August 2023, and only five articles were published in these 7 months of the year. Table 4 presents the main characteristics of the selected articles. A full list of the included articles with their main characteristics is presented in Additional file 2.

**Table 4:** Characteristics of included articles (*n* = 88)

Type of article	
Theoretical article	40 (45.5%)
Descriptive article	19 (21.6%)
Narrative review	18 (20.5%)
Systematic review	3 (3.4%)
Empirical study	44 (50.0%)
Protocols	4 (4.5%)
Field of study	
Health	71 (80.7%)
Healthcare and social services	42 (47.7%)
Health policy and systems	22 (25.0%)
Continuing education for healthcare professionals	7 (8.0%)
General	12 (13.6%)
Education	5 (5.7%)
KMb implementation context	
Canada	16 (18.2%)
Australia	9 (10.2%)
United States	12 (13.6%)
United Kingdom	5 (5.7%)
Europe	4 (4.5%)
Other*	8 (9.1%)
Not applicable**	34 (38.6%)
First authors’ country of affiliation	
Canada	31 (35.2%)
United States	23 (26.1%)
Australia	13 (14.8%)
United Kingdom	8 (9.1%)
Europe	7 (8.0%)
Other*	6 (6.8%)

* Countries from South America, the Caribbean, Africa, Southeast Asia, China and the Middle East

**Theoretical articles that did not implement a KMb strategy

The number of theoretical and empirical articles was relatively similar. Among the theoretical articles, 19 descriptive articles (21.6%) were aimed at describing a KMb strategy, a KMb infrastructure or a TMF related to a specific programme or context; 18 articles (20.5%) synthesized knowledge to propose a TMF (new or revised); and three articles conducted systematic reviews (3.4%).

The empirical articles category included studies with different methodological approaches (quantitative, qualitative, mixed methods). We will not report the details of the methodologies used, as this would result in a long list with few occurrences. The empirical articles can be divided into three categories: (1) studies that evaluated a TMF related to KMb (*n* = 16, 18.2%), (2) studies that evaluated a KMb strategy (*n* = 21, 23.9%) and (3) studies that evaluated both a KMb strategy and a TMF (*n* = 7, 8.0%).

Most articles were related to healthcare (*n* = 71, 80.7%). This field of study was divided into three subdomains. The healthcare and social services articles usually described or assessed a KMb strategy targeting health professionals’ practices in a variety of fields (for example, occupational therapy, dentistry, mental health, pharmacology, gerontology, nursing and so on). The health policy and systems articles usually described or assessed KMb strategies targeting decision-making processes, decision-makers or public health interventions and policies. The continuing education articles assessed training programmes for health professionals aimed at increasing knowledge and skills in a specific field. The articles in the general field described or discussed TMFs and KMb strategies that could be applied to multiple disciplines or contexts. Finally, the articles in the education field described or assessed a KMb strategy targeting education professionals.

Almost half of the articles (*n* = 42, 47.7%) studied KMB strategies implemented in only four countries: Canada, Australia, the United States and the United Kingdom. Countries in South America, the Caribbean, Africa, Asia, the Middle East, China and Europe were underrepresented (*n* = 8, 9.1%). The remaining 34 articles (38.6%) did not specify an implementation context and were mostly theoretical articles. Regarding the authors’ countries of affiliation, Canada, the United States, Australia and the United Kingdom were again the most represented countries, featuring in 85% of the articles (*n* = 75).

### What theories, models or frameworks exist in the literature to evaluate KMb strategies?

Several articles proposed a new TMF (*n* = 37, 42.0%), and some articles proposed a logic model specifically developed to evaluate their KMb strategy (*n* = 17, 19.3%). One-third of the articles used existing TMFs (*n* = 28, 31.8%). A few articles only referred to existing TMFs but did not use them to guide a KMb strategy evaluation (*n* = 6, 8.5%).

The identified TMFs were then categorized according to their theoretical approaches (adapted from Nilsen, [32]) (Table 5). Five articles used or proposed more than one TMF, and three TMFs could be classified in two categories. Several articles proposed or used a process model (*n* = 34, 38.6%) or an evaluation framework (*n* = 28, 31.8%); these were the two most frequently identified types of TMFs. Fewer articles proposed or used a logic model (*n* = 17, 19.3%), a determinant framework (*n* = 12, 13.6%) or a classic theory (*n* = 7, 8.0%). The TMFs most often identified in the articles were the RE-AIM framework (*n* = 5, 5.7%), the Knowledge-to-Action framework [9] (*n* = 4, 4.5%), the Theory of Planned Behavior [51] (*n* = 3, 3.4%) and the Expanded Outcomes framework for planning and assessing continuing medical education [52] (*n* = 3, 3.4%). In total, we identified 87 different TMFs in the 88 articles. Only nine TMFS were retrieved in more than one article.

**Table 5:** List of theories, models and frameworks identified in the selected articles

Theory, model or framework (source reference)	Identified in
Process Models	
ACT SMART implementation toolkit Combined analytical framework: science of using science and context matters	Tschida and Drahota 2023[53] Langer and Weyrauch 2020 [52]
Simplified framework of interventions to promote and integrate evidence	Colquhoun et al. 2014 [54]
Conceptual framework for assessing communities of practice in health policy	Bertone et al. 2013 [55]
Conceptual framework for patient-mediated KT interventions	Gagliardi et al. 2011 [56]
Conceptual model for continuing professional development (Kern, 1998)	Sargeant et al. 2011 [57]
Content, context and process model of strategic change (Pettigrew and Whipp, 1992)	Stetler et al. 2007 [58]
Contribution mapping: the three-phase process model	Kok and Schult 2012 [59]
Different strategies needed at different stages in the process of change (Grol, 2002)	Dadich 2010 [60]
Five steps of individual learning (Moore, 2008)	Sargeant et al. 2011 [57]
Framework for evaluating engagement intervention processes and outcomes	Brown and Bahri 2019 [61]
Framework for the dissemination and utilization of research	Dobbins et al. 2002 [62]
Framework of guideline implementability Framework to guide complex storytelling interventions	Gagliardi et al. 2012 [63] Brooks et al. 2022[64]
Iowa implementation for sustainability framework Knowledge translation planning template (KTPT) (Barwick, 2013)	Cullen et al. 2022[65] Labbé et al. 2020 [66]
Knowledge-to-action (KTA) framework (Graham et al., 2006)	Straus et al. 2010 [67] Graham et al. 2006 [9] Bennett et al. 2016 [68] Sargeant et al. 2011 [57]
Levels of use scale (Hall and Hord, 2015)	Brown and Rogers 2014 [69]
Operations triad model Participatory action research cycle	Talbott et al. 2023[70] Bennett et al. 2016 [68]
Plan-Do-Study-Act (PDSA) (Deming, 1986)	Sargeant et al. 2011 [57]
Population health planning knowledge-to-action model (Peirson and Rosella, 2015)	Rosella et al. 2018 [71]
Promotion of trauma-focused interventions informed by social cognitive theory	Couineau & Forbes, 2011 [72]
Six-step collaborative research utilization model	Dufault, 2004 [73]
Social impact framework	Beckett et al. 2018 [74]
Theory-based knowledge-transfer and exchange method of evaluation (KEME)	Kramer et al. 2013 [75]
The family systems nursing knowledge utilization model	Duhamel et al. 2015 [76]
The ladder of research use (Landry et al., 2003)	Brown and Rogers 2014 [69]
The modified pipeline model	Wimpenny et al. 2008 [77]
The revised conceptual framework of knowledge exchange (Ward et al., 2012)	Ward et al. 2012 [78] Grooten et al. 2020 [79]
The updated Stetler model of research utilization	Stetler 2001 [80]
The 4E conceptual framework: exposure, experience, expertise and embedding	Farkas et al. 2003 [43]
Why, whose, what, how framework for knowledge mobilizers (Ward, 2017)	Ward 2017 [81] Grooten et al. 2020 [79]
Evaluation frameworks	
Advancing research and clinical practice through close collaboration (ARCC) model	Levin et al. 2011 [82]
A model of the types of community impacts of research partnerships	Currie et al. 2005 [83]
Communities of practice (COP) evaluation model	Richard et al. 2014 [84]
Conceptual framework of CLAHRCs	Rycroft-Malone et al. 2013 [85]
Conceptual model of factors influencing effectiveness of knowledge exchange	Gagliardi et al. 2008 [86]
Diabetes evaluation framework for innovative national evaluations	Paquette-Warren et al. 2016 [87] Paquette-Warren et al. 2017 [88]
Dimensions of professional leaning communities (Hord, 2009)	Abbot et al. 2018 [89]
Evaluation dimensions and methodologies for technology-enabled KT Evaluation model of teachers’ knowledge sharing behaviour	Ho et al. 2004 [90] Yu et al. 2022[91]
Expanded outcomes framework for planning and assessing continuing medical education (Moore et al., 2009)	Moore et al. 2009 [52] Arora et al. 2017 [92] Sargeant et al. 2011 [57]
Framework for evaluating educational outcomes (Kirkpatrick, 1967; 1996)	Sargeant et al. 2011 [57] Smidt et al. 2009 [93]
Framework for assessing the impact of implementation of best-practice guidelines	Jeffs et al. 2013 [94]
Knowledge uptake and utilization tool (KUUT)	Skinner 2007 [95]
Knowledge translation planning template (KTPT) (Barwick, 2013)	Labbé et al. 2020 [66]
Measuring the impact of health research: an assessment tool	Lavis et al. 2003 [96]
Model of deliberative dialogues as a KTE strategy	Boyko et al. 2012 [97]
Modified Kirkpatrick’s framework (Barr, 2005)	Sargeant et al. 2011 [57]
Outcomes for implementation research	Proctor et al. 2011 [98]
RE-AIM framework (Glasgow et al., 1999)	Gainforth et al., 2015 [99] Glasgow et al. 2019 [100] Shelton et al. 2020[101] Bender et al. 2021[102] De la Garza et al. 2023[103]
Types of evaluation use	Alkin and Taut 2003 [104]
Working conceptual model for embedded implementation research	Varallyay et al. 2020 [105]
Determinant frameworks	
A model for collaborative working to facilitate KMb in public health	McCabe et al. 2015 [106]
A simplified framework of interventions to promote and integrate evidence	Colquhoun et al. 2014 [54]
Conceptual model of factors influencing effectiveness of knowledge exchange IDS-based conceptual framework for translating evidence into practice, policy and public health improvements	Gagliardi et al. 2008 [86] Gonzales et al. 2012[107]
Joint venture model of knowledge utilization (JVMKU)	Edgar 2006 [108]
PARIHS framework (Kitson et al., 1998)	Stetler et al. 2011 [109]
Professional learning evaluation 5-level framework (Guskey, 2014)	Abbot et al. 2018 [89]
Theoretical domains framework (TDF) (Michie et al. 2005)	Bennett et al. 2016 [68] Brennan et al. 2016 [110]
The knowledge translation and exchange framework for road safety TITO framework VEDMAP (value- and evidence-based decision-making and practice)	Hinchcliff et al. 2017 [111] Ye et al. 2022[112] Mfuso-Bengo et al., 2023
Classic theories	
Evidence-based practice confidence (EPIS) scale (Salbach et al., 2013)	Brangan et al. 2015 [113]
Innovation-decision process (Rogers, 2003)	Gainforth et al. 2015 [99]
Social cognitive theory (Bandura, 1997)	Bonetti et al. 2009 [114]
Stages of change model (Prochaska and DiClemente, 1985)	Buckley et al. 2003 [115]
Stages of change readiness and treatment eagerness scale (Miller and Tonnigan, 1996)	Buckley et al. 2003 [115]
Theory of planned behavior (TPB) (Ajzen, 1991)	Boyko et al. 2011 [116] Bonetti et al. 2009 [114] Imani-Nasab et al. 2017 [117]
Two-communities theory (Caplan, 1979)	Dwan et al. 2015 [118]
Logic Models	
Australian prevention partnership centre model	Haynes et al. 2020 [119]
Australian NSW Agency for Clinical Innovation – networks project logic framework CDC science impact framework	Haines et al. 2012 [120] Ko et al. 2019[121]
Center of Excellence for Training and Research translation’s evaluation framework	Leeman et al. 2012 [122]
CNODES’ knowledge translation logic model	Sketris et al. 2020 [49]
CO-OPS KTE platform’s logic model and evaluation plan Conceptual model for building programme sustainability in public health settings	Pettman et al. 2016 [123] Moreland-Russell et al. 2023
Evidence Informed Decision Making Network of the Caribbean (EvIDeNCe) initiative EVIPNet Europe M&E Framework: WHO Secretariat level logic model and EVIPNet Europe M&E Framework: country team/KTP level logic model	Yearwood et al. 2018 [124] Kuchenmüller et al. 2022
KT platform 4C programme theory	Thomson et al. 2019 [125]
Logic model of the Center of Research Excellence in Polycystic Ovary Syndrome	Garad et al., 2018 [126]
Policy liaison initiative (PLI) logic model	Brennan et al., 2016 [110]
Programme theory of The Remote Primary Health Care Manuals The institute for Work and Health Research impact model The logic model for the INSPIRE care model	Reddy et al. 2015 [127] Van Eerd et al. 2021[128] Yip et al. 2021[129]2023–12-14 12:10:00
The logic model of the evidence-based practice training programme	Guo et al. 2011 [130]
The SEA-ORCHID logical framework	McDonald et al. 2010 [131]

### What KMb strategies do the TMFs evaluate (activities, objectives, target audience)?

Thirty-eight articles reported using more than one activity in their KMb strategy. According to the ERIC compilation, “Train and educate stakeholders” activities were the most common, followed by “Develop stakeholder interrelationships” and “Use evaluative and iterative strategies”. Table 6 presents the various types of activities and the number of articles that referred to each.

**Table 6:** Types of KMb activities identified in the articles

KMb activities	Number of articles* (*n* = 88)
Train and educate stakeholders	46 (52.3%)
Develop stakeholder interrelationships	23 (26.1%)
Use evaluative and iterative strategies	17 (19.3%)
Provide interactive assistance	14 (15.9%)
Adapt and tailor to context	7 (8.0%)
Engage consumers	6 (6.8%)
`Change infrastructure	4 (4.5%)
Utilize financial strategies	1 (1.1%)
Support clinicians	1 (1.1%)

*Several articles identified many different activities in their KMb strategies

Of the 88 articles analysed, 18 (20.4%) did not specify a KMb objective. The remaining articles proposed one or more KMb strategy objectives. Specifically, 39 (36.4%) articles had one objective, 15 (17.0%) had two, three (3.4%) had three, and 13 (14.8%) had four or five. Table 7 presents the different types of objectives and the number of times they were identified.

**Table 7:** Types of KMb objectives identified in the articles

KMb objectives	Number of articles* (*n* = 88)
Expose – inform	17 (19.3%)
Change attitude – raise awareness	9 (10.2%)
Inform and influence decision-making	12 (13.6%)
Improve practices – increase expertise	30 (34.1%)
Foster collaboration	12 (13.6%)

*Some articles mentioned several objectives and others none

The target audiences for KMb strategies were clearly specified in half of the articles (*n* = 44, 50.0%). Generally, these were empirical articles that targeted specific professionals (*n* = 36, 40.9%) or decision-makers (*n* = 8, 9.1%). Just under one-third of the articles identified a broad target audience (for example, professionals and managers in the health system, a health organization) (*n* = 26, 29.5%). Finally, 18 articles (20.4%) did not specify a target audience for KMb; these were most often theoretical articles.

### What are the dimensions and components included in TMFs for evaluating KMb strategies?

**Fig. 2:**
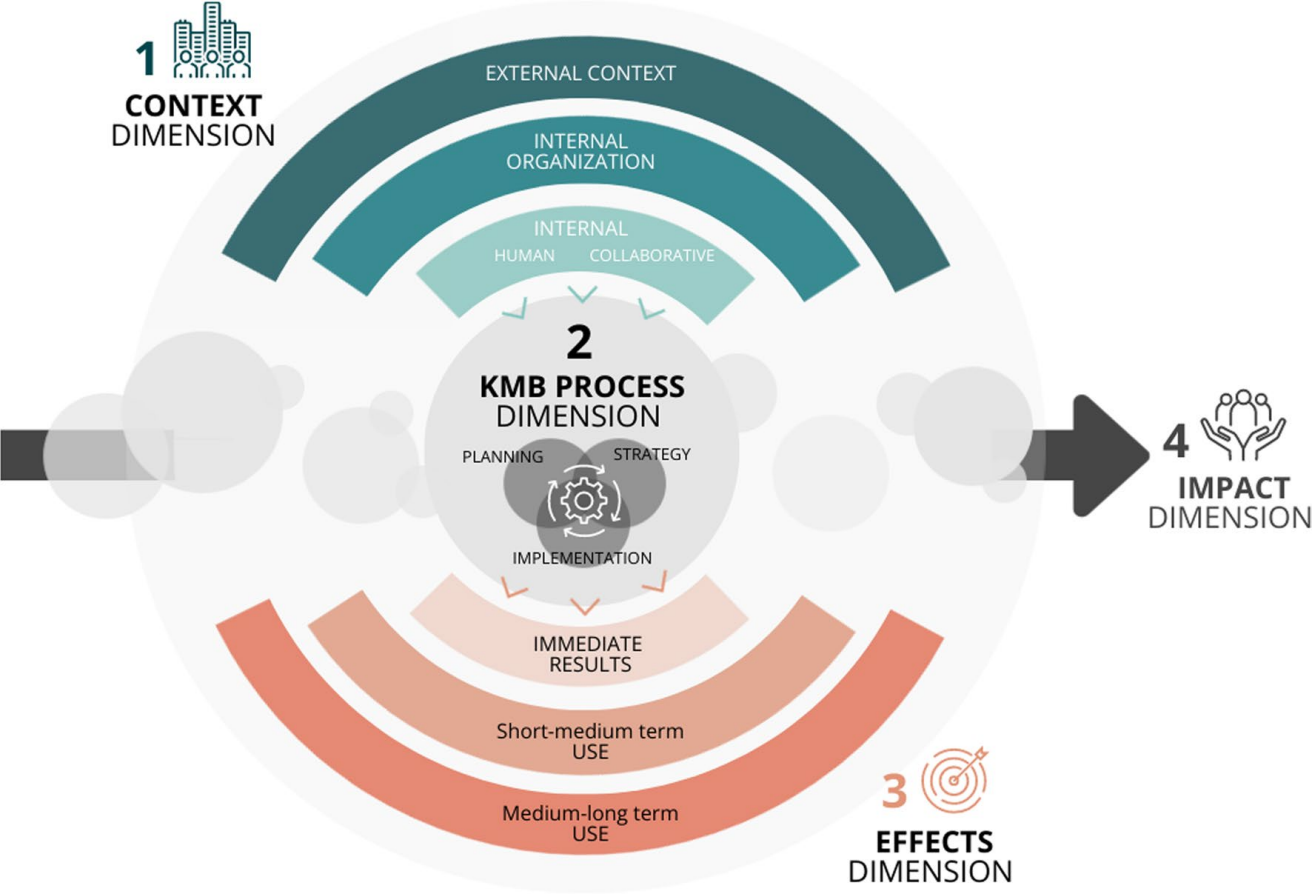
The main evaluation dimensions that emerged from the TMFs analysed

The analysis of the identified TMFs revealed many factors of interest relevant for the evaluation of KMb strategies. These specific components were inductively classified into four main dimensions: context, process, effects and impacts (Fig. 2). The context dimension refers to the assessment of the conditions in place when the KMb strategy is implemented. These include both the external (that is, sociopolitical, economic, environmental and cultural characteristics) and internal environments (that is, characteristics of organizations, individuals and stakeholder partnerships). These factors are understood to influence the selection and tailoring of a KMb strategy. The process dimension refers to the assessment of the planning, levels and mechanisms of implementation, as well as to the characteristics of the KMb strategy implemented. The effects dimension refers to the assessment of outcomes following the KMb strategy implementation. The potential effects vary depending on the strategy’s objectives and can be either the immediate results of the KMb strategy or short-, medium- and long-term outcomes. The conceptual gradation of effects was generally represented in a similar way in the TMFs analysed, but the temporality of effects could vary. A medium-term outcome in one study could be understood as a long-term outcome in another. However, the majority of authors group these effects into three categories (Gervais et al. 2016: p. 6): (1) short-term effects, measured by success of KMb strategy measured by success of KMb strategy (number of people reached, satisfaction, participation and so on); (2) medium-term effects linked to changes in individual attitude and the use of knowledge; and (3) the long-term effects that result from achieving the KMb objective (for example, improved practices and services, changed collective behaviour, sustainable use of knowledge).

Finally, the impacts dimension refers to the ultimate effects of KMb products or interventions on end users, as measured by the organization (Phipps et al. [36], p. 34). The evaluation of these ultimate effects can be measured by the integration of a promising practice into organizational routines, by the effects on service users or by the effects on the health and well-being of communities and society in general.

This gradation shows the importance of measuring effects at different points in time, to take account of the time they take to appear and their evolving nature (Gervais et al., 2016: p. 6).

Most of the articles presented the dimensions that should be evaluated, whereas the empirical articles presented the dimensions but also used them in practice to evaluate a KMb strategy. Only five articles (5.7%) did not mention specific dimensions that could be classified.

Table 8 presents both the number of articles that presented dimensions to be evaluated and the number of articles that evaluated them in practice. These results showed that the effects dimension was both the most often named and the most evaluated in practice. The other three dimensions (context, process, impacts), while quite often mentioned as relevant to assess, were less often evaluated in practice. For example, only five articles (5.7%) reported having assessed the impacts dimension.

**Table 8:** Number of articles that mentioned or evaluated the different dimensions

Dimensions	Number of articles that mentioned the dimensions (*n* = 88)	Number of articles that evaluated the dimensions (*n* = 88)
(1) Context	42 (47.7%)	15 (17.0%)
(2) Process	46 (52.3%)	16 (18.2%)
(3) Effects (immediate result; short-, medium- and long-term effects)	75 (85.2%)	32 (36.4%)
(4) Impacts (or benefits)	47 (53.4%)	5 (5.7%)

As previously mentioned, the components relevant for the evaluation of KMb strategies were extracted from the identified TMFs. Table 9 presents these components, which represent the more specific factors of interest for assessing context, process, effects and impacts.

**Table 9:** Dimensions, subdimensions and components for evaluating KMb strategies

Dimensions	Subdimensions	Components
(1) Context	External context	Supportive external environment Response to needs Accessibility to knowledge Priority given to knowledge
	Internal context – organization	Structural characteristics Organizational culture Available resources Networks and communications Implementation climate Leadership
	Internal context – individuals (target population)	Personal characteristics Attitudes (for example, receptivity, motivation) Beliefs (for example, perceived value and utility) Knowledge and skills (pre-strategy objective assessment) Perceived knowledge and skills (pre-strategy)
	Internal context – partnership and collaboration	Functioning of the partnership or collaboration Perceived quality of the partnership or collaboration Characteristics of the group of partners or collaborators
(2) Process	Planification	Presence of a KMb plan (intervention logic) Presence of an evaluation plan for the KMb strategy
	KMb strategy	Characteristics of the strategy (for example, types of activities) Characteristics of the content (for example, knowledge quality) Characteristics of those responsible for the KMb strategy
	Implementation	Level of participation Reason (motivation) for participation Participants’ attitudes and commitment Implementation fidelity Adaptation of the KMb strategy Implementation and evaluation follow-up
(3) Effects	Immediate results	Participants’ satisfaction Perceived learning Objective learning Sense of competence (self-efficacy) Change in beliefs and attitudes Intention to use knowledge
	Short- to medium-term effect of evidence use	Knowledge adoption Knowledge appropriation Knowledge application Decision-making support using acquired knowledge Intent to maintain knowledge use Collaboration development
	Medium- to long-term effect of evidence use	Effectiveness of knowledge use Development of competence Individual behaviour change Collective behavior change Organizational change Sustained knowledge use Updating (adaptation) of knowledge through practice Improved practices and services Sharing of acquired expertise Maintenance of the collaboration
(4) Impacts	Impacts or benefits of evidence use	Impacts on people receiving services Impacts on professionals Impacts on organizations, policies or systems Impacts on the community or population

## Discussion

Although often overlooked, the evaluation of KMb strategies is an essential step in guiding organizations seeking to determine whether the expected outcomes of their initiatives are being realized. Evaluation not only allows organizations to make adjustments if the initiatives are not producing the expected results, but also helps them to justify their funding of such initiatives. Evaluation is also essential if the KMb science is to truly inform KMb practice, such that the strategies developed are based on empirical data [30]. To make KMb evaluation more feasible, evaluation must be promoted and practices improved.

This scoping review meets the first objective of our project, which was to provide an overview of reference frameworks used or proposed for evaluating KM strategies, and to propose a preliminary version of a reference framework for evaluating KM strategies. Several key findings emerged from this scoping review:

### Proliferation of theories, models and frameworks, but few frequently used

We are seeing a proliferation of TMFs in KMb and closely related fields [132, 133]. Thus, the results of this scoping review support the argument that the conceptual and theoretical development of the field is outpacing its empirical development. Most of the reviewed articles (42.0%) proposed a new TMF rather than using existing ones. Furthermore, we identified relatively few empirical studies (50.0%) that focused on the evaluation of KMb strategies. Consequently, the TMFs used were poorly consolidated, which does not provide a solid empirical foundation to guide the evaluation of KMb strategies. Also, not all the TMFs proposed in the articles were specifically developed for evaluation; some were focused on KMb implementation processes. These may still provide elements to consider for evaluation, although they were not designed to propose specific indicators.

A scoping review published in 2018 identified 596 studies using 159 different KMb TMFs, 95 of which had been used only once [11]. Many authors reported that these are rarely reused and validated [11, 30, 33] and that it is important to test, refine and integrate existing ones [3, 31, 34, 133]. A clear, collective and consistent use of existing TMFs is recommended and necessary to advance KMb science and closely related fields [12, 31]. The systematic review by Strifler et al. [11] highlights the diversity of available TMFs and the difficulty users may experience when choosing TMFs to guide their KMb initiatives or evaluation process. Future work should focus on the development of tools to better support users of TMFs, especially those working in organizations. By consolidating a large number of TMFs, the results of this scoping review contribute to these efforts.

### The importance of improving evaluation practices for complex multifaceted KMb strategies

Another noteworthy finding was the emphasis on the evaluation of strategies focused on education and professional training for practice improvement (52.3%). Relatively few of the reviewed articles looked at, for example, the evaluation of KMb strategies aimed at informing or influencing decision-making (13.6%), or KMb strategies targeting decision-makers (9.1%). These results reaffirm the importance of conducting more large-scale evaluations of complex and multifaceted KMb strategies. These involve a greater degree of interaction and engagement, are composed of networks of multiple actors, mobilize diverse sources of knowledge and have simultaneous multilevel objectives [19, 134].

The fact that some KMb strategies are complex interventions implemented in complex contexts [134] presents a significant and recurring challenge to their evaluation. Methodological designs, approaches and tools are often ill-suited to capture the short-, medium- and long-term outcomes of KMb strategies, as well as to identify the mechanisms by which these outcomes were produced in a specific context. It is also difficult to link concrete changes in practice and decision-making to tangible longer-term impacts at the population level. Moreover, these impacts can take years to be achieved [36] and can be influenced by several other factors in addition to KMb efforts [2, 19, 24]. Comprehensive, dynamic and flexible evaluation approaches [135–137] using mixed methods [20] appear necessary to understand why, for whom, how, when and in what context KMb strategies achieve their objectives [2, 21, 25]. For instance, realist evaluation, which belongs to theory-based evaluation, may be an approach that addresses issues of causality without sacrificing complexity [134, 138, 139]. This evaluation approach aims to identify the underlying generative mechanisms that can explain how the outcomes were generated and what characteristics of the context affected, or not, those mechanisms. This approach is used to test and refine theory about how interventions with a similar logic of action actually work [139].

### Large heterogeneity of methodologies used in empirical studies

Despite the growth of the KMb field, a recurring issue is the relatively limited number of high-quality studies that evaluate KMb outcomes and impacts. This observation is shared by many of the authors of our scoping articles [2, 22–25]. Only a limited number of empirical articles met the selection criteria (*n* = 44/88) in this scoping review. Synthesizing these studies is challenging due to the diversity of research designs used and the large number of potential evaluation components identified. In addition, most of the identified studies used TMFs and measurement tools that were not validated [20, 29] and that were specifically developed for their study [16, 25, 140]. Moreover, these studies did not describe the methods used to justify their choice of evaluation dimensions and components [25], which greatly hinders the ability to draw inferences and develop generalizable theories through replication in similar studies [110, 140–143]. The lack of a widely used evaluation approach across the field is therefore an important issue [16, 20] also highlighted by this scoping review.

Our aim in this review was not to identify specific indicators or measurement tools (for example, questionnaires) for assessing KMb strategies, but rather to describe dimensions and component of TMFs used for KMb evaluation. However, a recent scoping review [144] looked at measurement tools and revealed that only two general potential tools have been identified to assess KMb activities in any sector or organization: the *Level of Knowledge Use Survey* (LOKUS) [145] and the *Knowledge Uptake and Utilization Tool* (KUUT) [95]. The authors also assert the importance of developing standardized tools and evaluation processes to facilitate comparison of KMb activities’ outcomes across organizations [144].

### Lack of description and reporting of KMb strategies and evaluation

Another important finding from this review was the sparsity of descriptions of KMb strategies in the published articles. In general, the authors provided little information on the operationalization of their KMb strategies (for example, objectives, target audiences, details of activities implemented, implementation context, expected effects). The KMb strategy objectives and the implemented activities should be carefully selected and empirically, theoretically or pragmatically justified before the evaluation components and specific indicators can be determined [146].

To improve consistency in the field and to contribute to the development of KMb science, many authors reported the need to better describe and report KMb strategies and their context [8, 54, 146–150]. KMb strategies are often inconsistently labelled across studies, poorly described and rarely justified theoretically [146, 150, 151]. It was not possible in this scoping review to associate the evaluation components to be used with the objectives and types of KMb strategies, as too much information was missing in the articles. Over the past 10 years, several guidelines have been proposed to improve the reporting of interventions such as KMb strategies: the “Workgroup for Intervention Development and Evaluation Research (WIDER) recommendations checklist” [147], the “Standards for Reporting Implementation Studies (StaRI)” [150] and the “Template for Intervention Description and Replication (TIDieR)” [152]. These guidelines should be used more often to enhance the reporting of KMb strategies and help advance the field [153].

## Implications for future research

This scoping review provides an overview of potential factors of interest for assessing the context, process, effects and impacts of a KMb strategy. It also proposes a preliminary inventory of potential dimensions and components to consider when planning the evaluation of a KMb strategy. Given the broad spectrum of factors of interest identified across studies, not all of them can be assessed in every context. Rather, they should be targeted according to the objectives of the evaluation, the nature of the KMb strategy and the resources available to conduct the evaluation. Thus, this inventory should not be understood as a prescriptive, normative and exhaustive framework, but rather as a toolbox to identify the most relevant factors to include in the evaluation of a given KMB strategy, and to address a need often expressed by organizations wishing to evaluate their KMb efforts.

Additional work is needed to validate and operationalize these dimensions, to identify relevant measurement tools related to the different components and to see how this inventory could support KMb evaluation practices in organizations.

This scoping review is the first stage of a larger research project aimed at improving organizations’ capacity to evaluate their KMb initiatives by developing an integrative, interdisciplinary and easy-to-use reference framework. In the second phase of the project, the relevance and clarity of the evaluation dimensions identified in the scoping review will be validated through a Delphi study with KMb specialists and researchers. The enriched framework will then be pilot tested in two organizations carrying out and evaluating KMb strategies, to adapt the framework to their needs and to further clarify how the dimensions can be measured in practice. In this third phase, guidance will be provided to help organizations adopt the framework and its support kit. The aim of the project is to go beyond proposing a theoretical framework, and to help build organizations’ capacity to evaluate KT strategies by proposing tools adapted to their realities.

## Review limitations

Some limitations of this scoping review should be acknowledged. First, given the numerous different terms used to describe and conceptualize the science of using evidence, it is possible that our search strategy did not capture all relevant publications. However, to limit this risk, we manually searched the reference lists of the selected articles. Second, the literature search was limited to articles published in English or French, and the articles were mostly from high-income countries (for example, North America); therefore, the application of the identified concepts in this scoping review to other contexts should be further explored.

In addition, the search strategy focused on scientific publications to assess progress made in the field of knowledge mobilization strategy evaluation. The grey literature was not examined. It should be considered in future research to complete the overview of evaluation needs in the field of knowledge mobilization.

Finally, the paucity of information in the articles sometimes made it difficult to classify the TMFs according to the taxonomies [32, 44], which may have led to possible misinterpretation. However, to limit the risk of errors, the categorization was performed by two reviewers and validated by a third in cases of uncertainty.

## Conclusions

Given the increasing demand from organizations for the evaluation of KMb strategies, along with the poorly consolidated KMb research field, a scoping review was needed to identify the range, nature and extent of the literature. This scoping review enabled us to synthesize the breadth of the literature, provide an overview of the many theories, models and frameworks used, and identify and categorize the potential dimensions and components to consider when evaluating KMb initiatives. This scoping review is part of a larger research project, in which the next steps will be to validate the integrative framework and develop a support kit to facilitate its use by organizations involved in KMb.

### Supplementary Information

**Additional file 1.** Keywords and search strategy.

**Additional file 2.** Summary of included articles.

## Data Availability

The dataset supporting the conclusions of this article is included within the article and its additional files.

## Abbreviations

KMb: Knowledge mobilization
TMFs: Theories, models, and frameworks
