# Supplementary material for: A scoping review of theories, models and frameworks used or proposed to evaluate knowledge mobilization strategies
Source: Health Res Policy Syst. 2024 Jan 10;22:8. doi: 10.1186/s12961-023-01090-7 (PMC10777658; doi:10.1186/s12961-023-01090-7)
Supplement: Supplementary file 1 — Additional file 1. Keywords and search strategy. [file 12961_2023_1090_MOESM1_ESM.pdf]

ADDITIONAL FILE 1 – Keywords and search strategy

KMB	EVALUATION	TMF
Research knowledge<br>Knowledge transfer<br>Knowledge translation<br>Knowledge utilisation<br>Knowledge utilization<br>Knowledge sharing<br>Knowledge mobilisation<br>Knowledge mobilization<br>Knowledge dissemination<br>Knowledge diffusion<br>Knowledge exchange<br>Knowledge uptake<br>Knowledge use<br>Evidence uptake<br>Evidence use<br>Evidence utilisation<br>Evidence utilization<br>Research uptake<br>Research transfer<br>Research translation<br>Research utilisation<br>Research utilization<br>Research dissemination<br>Research sharing<br>Research mobilisation<br>Research mobilization<br>Research use<br>Research diffusion<br>Evidence-based practice<br>Research-based evidence<br>Implementation science<br>Research implementation<br>Evidence implementation	Evaluat*<br>Measur*<br>Impact*<br>Outcome*<br>Assess*<br>Apprais*<br>Indicator*	Framework*<br>Model*<br>Method*<br>Guide*<br>Theor*

Appendix 1 Search Strategy (2000-2023)

BDD	Recherche	Trouvés
Medline	1. (("research knowledge" or "knowledge transfer" or "knowledge translation" or "knowledge utilisation" or "knowledge utilization" or "knowledge sharing" or "knowledge mobilisation" or "knowledge mobilization" or "knowledge dissemination" or "knowledge diffusion" or "knowledge exchange" or "knowledge uptake" or "knowledge use" or "evidence uptake" or "evidence use" or "evidence utilisation" or "evidence utilization" or "research uptake" or "research transfer" or "research translation" or "research utilisation" or "research utilization" or "research dissemination" or "research sharing" or "research mobilisation" or "research mobilization" or "research use" or "research diffusion" or "evidence-based practice*" or "research-based evidence*" or **Implementation Science/ or ("implementation science" or "research implementation" or "evidence implementation"*").ab,ti. AND (evaluat* or measur* or impact* or outcome* or assess* or apprais* or indicator*).ab,ti. AND ((framework or model or method or guide or guideline or theory).ti. OR (framework* or model* or method* or guide* or theor*).kw.)) *AND 2000:2024.(sa_year).)*	1800
PsycInfo	1. ((((title: ("research-based evidence"))) *OR* ((abstract: ("research-based evidence")))) *OR* (((title: ("evidence-based practice"))) *OR* ((abstract: ("evidence-based practice")))) *OR* (((((title: ("research diffusion")))) *OR* (((abstract: ("research diffusion"))))) *OR* ((((title: ("research use")))) *OR* (((abstract: ("research use"))))) *OR* ((((title: ("research mobilization")))) *OR* (((abstract: ("research mobilization"))))) *OR* ((((title: ("research mobilisation")))) *OR* (((abstract: ("research mobilisation"))))) *OR* ((((title: ("research sharing")))) *OR* (((abstract: ("research sharing"))))) *OR* ((((title: ("research dissemination")))) *OR* (((abstract: ("research dissemination"))))) *OR* ((((title: ("research utilization")))) *OR* (((abstract: ("research utilization"))))) *OR* ((((title: ("research utilisation")))) *OR* (((abstract: ("research utilisation"))))) *OR* ((((title: ("research translation")))) *OR* (((abstract: ("research translation"))))) *OR* ((((title: ("research transfer")))) *OR* (((abstract: ("research transfer"))))) *OR* ((((title: ("research uptake")))) *OR* (((abstract: ("research uptake"))))) *OR* ((((title: ("evidence utilization")))) *OR* (((abstract: ("evidence utilization"))))) *OR* ((((title: ("evidence utilisation")))) *OR* (((abstract: ("evidence utilisation"))))) *OR* ((((title: ("evidence use")))) *OR* (((abstract: ("evidence use"))))) *OR* ((((title: ("evidence uptake")))) *OR* (((abstract: ("evidence uptake"))))) *OR* ((((title: ("knowledge use")))) *OR* (((abstract: ("knowledge use"))))) *OR* ((((title: ("knowledge uptake")))) *OR* (((abstract: ("knowledge uptake"))))) *OR* ((((title: ("knowledge exchange")))) *OR* (((abstract: ("knowledge exchange"))))) *OR* ((((title: ("knowledge diffusion")))) *OR* (((abstract: ("knowledge diffusion"))))) *OR* ((((title: ("knowledge dissemination")))) *OR* (((abstract: ("knowledge dissemination"))))) *OR* ((((title: ("knowledge mobilization")))) *OR* (((abstract: ("knowledge mobilization"))))) *OR* ((((title: ("knowledge mobilisation")))) *OR* (((abstract: ("knowledge mobilisation"))))) *OR* ((((title: ("knowledge sharing")))) *OR* (((abstract: ("knowledge	2025

	sharing"))))) *OR* ((((title: ("knowledge utilization")))) *OR* (((abstract: ("knowledge utilization"))))) *OR* ((((title: ("knowledge utilisation")))) *OR* (((abstract: ("knowledge utilisation"))))) *OR* ((((title: ("knowledge translation")))) *OR* (((abstract: ("knowledge translation"))))) *OR* ((((title: ("knowledge transfer")))) *OR* (((abstract: ("knowledge transfer"))))) *OR* (((title: ("implementation science"))) *OR* ((abstract: ("implementation science"))))) *OR* (((title: ("research implementation"))) *OR* ((abstract: ("research implementation")))) *OR* (((title: ("evidence implementation"))) *OR* ((abstract: ("evidence implementation"))))) *AND* ((((title: (indicator*))) *OR* ((abstract: (indicator*)))) *OR* (((title: (apprais*))) *OR* ((abstract: (apprais*)))) *OR* (((title: (assess*))) *OR* ((abstract: (assess*)))) *OR* (((title: (outcome*))) *OR* ((abstract: (outcome*)))) *OR* (((title: (impact*))) *OR* ((abstract: (impact*)))) *OR* (((title: (measur*))) *OR* ((abstract: (measur*)))) *OR* (((title: (evaluat*))) *OR* ((abstract: (evaluat*))))) *AND* ((((KEYWORDS: (guide*))) *OR* (((KEYWORDS: (method*))) *OR* (((KEYWORDS: (theor*))) *OR* (((KEYWORDS: (model*))) *OR* (((KEYWORDS: (framework*))))) *AND* Year: 2000 *To* 2024	
ERIC	1. ((((ti("knowledge transfer") OR ab("knowledge transfer")) OR (ti("knowledge translation") OR ab("knowledge translation")) OR (ti("knowledge utilisation") OR ab("knowledge utilisation")) OR (ti("knowledge utilization") OR ab("knowledge utilization")) OR (ti("knowledge sharing") OR ab("knowledge sharing")) OR (ti("knowledge mobilisation") OR ab("knowledge mobilisation")) OR (ti("knowledge mobilization") OR ab("knowledge mobilization")) OR (ti("knowledge dissemination") OR ab("knowledge dissemination")) OR (ti("knowledge diffusion") OR ab("knowledge diffusion")) OR (ti("knowledge diffusion") OR ab("knowledge diffusion")) OR (ti("knowledge exchange") OR ab("knowledge exchange")) OR (ti("knowledge uptake") OR ab("knowledge uptake")) OR (ti("knowledge use") OR ab("knowledge use")) OR (ti("evidence uptake") OR ab("evidence uptake")) OR (ti("evidence use") OR ab("evidence use")) OR (ti("evidence utilisation") OR ab("evidence utilisation")) OR (ti("evidence utilization") OR ab("evidence utilization")) OR (ti("research uptake") OR ab("research uptake")) OR (ti("research transfer") OR ab("research transfer")) OR (ti("research translation") OR ab("research translation")) OR (ti("research utilisation") OR ab("research utilisation")) OR (ti("research utilization") OR ab("research utilization")) OR (ti("research dissemination") OR ab("research dissemination")) OR (ti("research sharing") OR ab("research sharing")) OR (ti("research mobilisation") OR ab("research mobilisation")) OR (ti("research mobilization") OR ab("research mobilization")) OR (ti("research use") OR ab("research use")) OR (ti("research diffusion") OR ab("research diffusion")) OR (ti("evidence-based practice") OR ab("evidence-based practice")) OR (ti("research-based evidence") OR ab("research-based evidence")) OR (ti("implementation science") OR ti("research implementation") OR ti("evidence implementation") OR ab("implementation science") OR ab("research implementation") OR ab("evidence implementation"))) AND (ti(evaluat* OR measur* OR impact* OR outcome* OR assess* OR apprais* OR indicator*) OR ab(evaluat* OR measur* OR impact* OR outcome* OR assess* OR apprais* OR indicator*)) AND (ti(framework OR model OR	1014

	method OR theory OR guide OR guideline) OR su(framework* OR model* OR method* OR theor* OR guide*))) AND pd(20000101-20241231))	
Sociological Abstracts	1. ((((ti("knowledge transfer") OR ab("knowledge transfer")) OR (ti("knowledge translation") OR ab("knowledge translation")) OR (ti("knowledge utilisation") OR ab("knowledge utilisation")) OR (ti("knowledge utilization") OR ab("knowledge utilization")) OR (ti("knowledge sharing") OR ab("knowledge sharing")) OR (ti("knowledge mobilisation") OR ab("knowledge mobilisation")) OR (ti("knowledge mobilization") OR ab("knowledge mobilization")) OR (ti("knowledge dissemination") OR ab("knowledge dissemination")) OR (ti("knowledge diffusion") OR ab("knowledge diffusion")) OR (ti("knowledge diffusion") OR ab("knowledge diffusion")) OR (ti("knowledge exchange") OR ab("knowledge exchange")) OR (ti("knowledge uptake") OR ab("knowledge uptake")) OR (ti("knowledge use") OR ab("knowledge use")) OR (ti("evidence uptake") OR ab("evidence uptake")) OR (ti("evidence use") OR ab("evidence use")) OR (ti("evidence utilisation") OR ab("evidence utilisation")) OR (ti("evidence utilization") OR ab("evidence utilization")) OR (ti("research uptake") OR ab("research uptake")) OR (ti("research transfer") OR ab("research transfer")) OR (ti("research translation") OR ab("research translation")) OR (ti("research utilisation") OR ab("research utilisation")) OR (ti("research utilization") OR ab("research utilization")) OR (ti("research dissemination") OR ab("research dissemination")) OR (ti("research sharing") OR ab("research sharing")) OR (ti("research mobilisation") OR ab("research mobilisation")) OR (ti("research mobilization") OR ab("research mobilization")) OR (ti("research use") OR ab("research use")) OR (ti("research diffusion") OR ab("research diffusion")) OR (ti("evidence-based practice") OR ab("evidence-based practice")) OR (ti("research-based evidence") OR ab("research-based evidence")) OR (ti("implementation science") OR ti("research implementation") OR ti("evidence implementation") OR ab("implementation science") OR ab("research implementation") OR ab("evidence implementation"))) AND (ti(evaluat* OR measur* OR impact* OR outcome* OR assess* OR apprais* OR indicator*) OR ab(evaluat* OR measur* OR impact* OR outcome* OR assess* OR apprais* OR indicator*)) AND (ti(framework OR model OR method OR theory OR guide OR guideline) OR su(framework* OR model* OR method* OR theor* OR guide*))) AND pd(20000101-20241231))	554
Dissertation and thesis	1. ((((ti("knowledge transfer") OR ab("knowledge transfer")) OR (ti("knowledge translation") OR ab("knowledge translation")) OR (ti("knowledge utilisation") OR ab("knowledge utilisation")) OR (ti("knowledge utilization") OR ab("knowledge utilization")) OR (ti("knowledge sharing") OR ab("knowledge sharing")) OR (ti("knowledge mobilisation") OR ab("knowledge mobilisation")) OR (ti("knowledge mobilization") OR ab("knowledge mobilization")) OR (ti("knowledge dissemination") OR ab("knowledge dissemination")) OR (ti("knowledge diffusion") OR ab("knowledge diffusion")) OR (ti("knowledge diffusion") OR ab("knowledge diffusion")) OR (ti("knowledge exchange") OR ab("knowledge exchange")) OR (ti("knowledge uptake") OR ab("knowledge uptake")) OR (ti("knowledge use") OR ab("knowledge use")) OR (ti("evidence uptake") OR ab("evidence uptake"))	13

		OR (ti("evidence use") OR ab("evidence use")) OR (ti("evidence utilisation") OR ab("evidence utilisation")) OR (ti("evidence utilization") OR ab("evidence utilization")) OR (ti("research uptake") OR ab("research uptake")) OR (ti("research transfer") OR ab("research transfer")) OR (ti("research translation") OR ab("research translation")) OR (ti("research utilisation") OR ab("research utilisation")) OR (ti("research utilization") OR ab("research utilization")) OR (ti("research dissemination") OR ab("research dissemination")) OR (ti("research sharing") OR ab("research sharing")) OR (ti("research mobilisation") OR ab("research mobilisation")) OR (ti("research mobilization") OR ab("research mobilization")) OR (ti("research use") OR ab("research use")) OR (ti("research diffusion") OR ab("research diffusion")) OR (ti("evidence-based practice") OR ab("evidence-based practice")) OR (ti("research-based evidence") OR ab("research-based evidence")) OR (ti("implementation science") OR ti("research implementation") OR ti("evidence implementation") OR ab("implementation science") OR ab("research implementation") OR ab("evidence implementation"))) AND (ti(evaluat* OR measur* OR impact* OR outcome* OR assess* OR apprais* OR indicator*) OR ab(evaluat* OR measur* OR impact* OR outcome* OR assess* OR apprais* OR indicator*)) AND (ti(framework OR model OR method OR theory OR guide OR guideline) OR su(framework* OR model* OR method* OR theor* OR guide*))) AND pd(20000101-20241231))	
Érudit	1.	(Titre, résumé, mots-clés : ("transfert des connaissances" OU "utilisation des connaissances" OU "partage des connaissances" OU "mobilisation des connaissances" OU "diffusion des connaissances" OU "utilisation de la recherche" OU "diffusion de la recherche" OU "pratique fondée sur les preuves" OU "pratiques fondées sur les preuves" OU "research knowledge" OU "knowledge transfer" OU "knowledge translation" OU "knowledge utilisation" OU "knowledge utilization" OU "knowledge sharing" OU "knowledge mobilisation" OU "knowledge mobilization" OU "knowledge dissemination" OU "knowledge diffusion" OU "knowledge exchange" OU "knowledge uptake" OU "knowledge use" OU "evidence uptake" OU "evidence use" OU "evidence utilisation" OU "evidence utilization" OU "research uptake" OU "research transfer" OU "research translation" OU "research utilisation" OU "research utilization" OU "research dissemination" OU "research sharing" OU "research mobilisation" OU "research mobilization" OU "research use" OU "research diffusion" OU "evidence-based practice" OU "research-based evidence" OU "implementation science" OU "research implementation" OU "evidence implementation" OU "science de l'implantation")) ET (Titre, résumé, mots-clés : (evaluat* OU measur* OU impact* OU outcome* OU assess OU apprais* OU indicator* OU mesur* OU effet* OU résultat* OU évaluer OU indicateur*)) ET (Titre, résumé, mots-clés : (framework* OU model* OU method* OU guide* OU theor*)) ET (Publié entre 2000 et 2023) ET (Fonds : Érudit)	63
Cairn	1.	(Titre ("transfert des connaissances" or "utilisation des connaissances" or "partage des connaissances" or "mobilisation des connaissances" or "diffusion des connaissances" or "utilisation de la recherche" or "diffusion de la recherche" or "pratique fondée sur les preuves" or "pratiques fondées sur les preuves" or "research knowledge" or "knowledge transfer" or "knowledge translation" or "knowledge	37

	utilisation" or "knowledge utilization" or "knowledge sharing" or "knowledge mobilisation" or "knowledge mobilization" or "knowledge dissemination" or "knowledge diffusion" or "knowledge exchange" or "knowledge uptake" or "knowledge use" or "evidence uptake" or "evidence use" or "evidence utilisation" or "evidence utilization" or "research uptake" or "research transfer" or "research translation" or "research utilisation" or "research utilization" or "research dissemination" or "research sharing" or "research mobilisation" or "research mobilization" or "research use" or "research diffusion" or "evidence-based practice" or "research-based evidence" OU" implementation science" OU "research implementation" OU "evidence implementation" OU "science de l'implantation") ET Titre (evaluat* OU measur* OU impact* OU outcome* OU assess OU apprais* OU indicator* OU mesur* OU effet* OU résultat* OU évaluer OU indicateur*) ET Titre (framework* OU model* OU method* OU guide* OU theor*))<br>2. Résumé ("transfert des connaissances" or "utilisation des connaissances" or "partage des connaissances" or "mobilisation des connaissances" or "diffusion des connaissances" or "utilisation de la recherche" or "diffusion de la recherche" or "pratique fondée sur les preuves" or "pratiques fondées sur les preuves" or "research knowledge" or "knowledge transfer" or "knowledge translation" or "knowledge utilisation" or "knowledge utilization" or "knowledge sharing" or "knowledge mobilisation" or "knowledge mobilization" or "knowledge dissemination" or "knowledge diffusion" or "knowledge exchange" or "knowledge uptake" or "knowledge use" or "evidence uptake" or "evidence use" or "evidence utilisation" or "evidence utilization" or "research uptake" or "research transfer" or "research translation" or "research utilisation" or "research utilization" or "research dissemination" or "research sharing" or "research mobilisation" or "research mobilization" or "research use" or "research diffusion" or "evidence-based practice" or "research-based evidence" OU "implementation science" OU "research implementation" OU "evidence implementation" OU "science de l'implantation") ET Résumé (evaluat* OU measur* OU impact* OU outcome* OU assess OU apprais* OU indicator* OU mesur* OU effet* OU résultat* OU évaluer OU indicateur*) ET Résumé (framework* OU model* OU method* OU guide* OU theor*))	
SOUS-TOTAL	Incluant les doublons	5506
TOTAL	Sans doublons	4725
