# Supplementary material for: A scoping review of theories, models and frameworks used or proposed to evaluate knowledge mobilization strategies
Source: Health Res Policy Syst. 2024 Jan 10;22:8. doi: 10.1186/s12961-023-01090-7 (PMC10777658; doi:10.1186/s12961-023-01090-7)
Supplement: Supplementary file 2 — Additional file 2. Summary of included articles. [file 12961_2023_1090_MOESM2_ESM.pdf]

**Appendix 2: List of included articles and charting of their analyzed characteristics**

Legend

Country
- Country: Where the KM strategy takes place
- (Country): Where the first author comes from

Field
- 1-1: healthcare, care and services
- 1-2: healthcare, health policy and systems
- 1-3: healthcare, continuing education for healthcare professionals
- 2: general field of KM
- 3: education

Article type
- E: experimental
- T: theoretical
- S: systematic review

Article aims
- 1: assess framework
- 2: assess KM strategy
- 3: synthesis
- 4: describe

Theory, Model, Framework
- P: Process framework
- D: determinant framework
- E: evaluation framework
- C: classic theory
- L: logic model

KM strategy type
- 1: Train and educate stakeholders
- 2: Develop stakeholder interrelationships
- 3: Use evaluative and iterative strategies
- 4: Provide interactive assistance
- 5: Adapt dans tailor to context
- 6: Engage consumers
- 7: Change infrastructure
- 8: Utilize financial strategies
- 9: Support clinicians
- - : no specified type of strategy

KM strategy aims
- I: inform
- A: change attitude
- D: influence decision-making
- P: improve practices
- C: foster collaboration
- M: multiple aim
- - : no specified aim

KM strategy target
- 1: target specified
- 2: expected target
- 3: no specified target

Evaluation components categories
- C: context
- P: process
- O: outcomes
- O*: outcomes (incomplete evaluation)[1]
- I: impact

[1]This article mentioned some outcomes components without assessing them in the study (ex.: assess only short-term outcomes but not mid- and long-terms outcomes)

| Authors (year) | Title | Journal | Country | Field | Article type | Article aim | Theory Model Framework | KM strategy type | KM strategy aim | KM strategy target | Components mentionned | Components evaluated |
|---|---|---|---|---|---|---|---|---|---|---|---|---|
| Abbot et al., (2018) | Evaluating the Effectiveness and Functionality of Professional Learning Communities in Adult ESL Programs | *TESL Canada Journal* | Canada (Canada) | 3 | E | 2 | D,E | 1,6 | I, P | 1 | O | O |
| Alkin & Taut (2003) | Unbundling Evaluation Use | Studies in Educational Evaluation | N/A (USA) | 3 | T | 3 | E | - | - | 3 | C,P,O | - |
| Arora et al., (2017) | Project ECHO: A Telementoring Network Model for Continuing Professional Development | JCEHP : Journal of Continuing Educiation in the Health Professions | USA (USA) | 1-3 | T | 4 | E | 1,2,3,5 | P | 1 | P,O,I | - |
| Beckett et al., (2018) | Embracing complexity and uncertainty to create impact: exploring the processes and transformative potential of co-produced research through development of a social impact model | Health Research Policy and Systems | UK, Canada (UK) | 1-2 | T | 3 | P | 7 | D | 2 | O,I | - |
| Bender et al (2021) | The Asthma Toolkit Bootcamp to Improve Rural Primary Care for Pediatric Asthma | The Journal of Allergy and Clinical Immunology: In Practice | USA (USA) | 1-1 | E | 2 | E | 1 | D | 1 | P,O | P,O |
| Bennett et al. (2016) | Building capacity for knowledge translation in occupational therapy: learning through participatory action research | BMC Medical Education | Australia (Australia) | 1-1 | E | 2 | P,D | 1,2,3 | P | 1 | C,P,O | C,P,O* |

| Authors (year) | Title | Journal | Country | Field | Article type | Article aim | Theory Model Framework | KM strategy type | KM strategy aim | KM strategy target | Components mentionned | Components evaluated |
|---|---|---|---|---|---|---|---|---|---|---|---|---|
| Berthone et al. (2013) | Assessing communities of practice in health policy: a conceptual framework as a first step towards empirical research | Health Research Policy and Systems | N/A (Belgium) | 1-2 | T | 3 | P | 1 | P | 2 | C,P,O,I | - |
| Bonetti et al. (2009) | Knowledge May Not Be the Best Target for Strategies. to Influence Evidence-Based Practice: Using Psychological Models to Understand RCT Effects | International journal of behavioral medicine | UK (Scotland) | 1-1 | E | 2 | C | 1 | D, P | 1 | O | O |
| Boyko et al. (2011) | Reliability of a tool for measuring theory of planned behaviour constructs for use in evaluating research use in policymaking | Health Research Policy and Systems | Canada (Canada) | 1-2 | E | 1 | C | 1 | D | 2 | C,O | C,O |
| Boyko et al. (2012) | Deliberative dialogues as a mechanism for knowledge translation and exchange in health systems decision-making | Social Science & Medicine | N/A (Canada) | 1-2 | S | 3 | D | 1,2,3,4,5,7,9 | I, D | 2 | O,I | - |
| Brangan et al. (2015) | Impact of an Evidence-Based Practice Course on Occupational Therapist's Confidence Levels and Goals | Occupational Therapy In Health Care | Ireland (Ireland) | 1-1 | E | 2 | C | 1 | A, P | 1 | O | O |

| Authors (year) | Title | Journal | Country | Field | Article type | Article aim | Theory Model Framework | KM strategy type | KM strategy aim | KM strategy target | Components mentionned | Components evaluated |
|---|---|---|---|---|---|---|---|---|---|---|---|---|
| Brennan et al. (2016) | Design and formative evaluation of the Policy Liaison Initiative: a long-term knowledge translation strategy to encourage and support the use of Cochrane systematic reviews for informing health policy | Evidence & Policy | Australia (Australia) | 1-2 | E | 2 | E | 1 | D | 1 | C,O,I | C,O* |
| Brooks et al., (2022) | A framework to guide storytelling as a knowledge translation intervention for health promoting behaviour change | Implementation science communication | Canada (Canada) | 2 | T | 4 | P | 1 | 1 | 3 | P | - |
| Brown & Rogers (2014) | Measuring the effectiveness of knowledge creation as a means of facilitating evidence-informed practice in early years settings in one London borough | London Review of Education | England (England) | 3 | E | 2 | C | 1 | P | 1 | O | O |
| Brown & Bahri (2019) | Engagement of patients and healthcare professionals in regulatory pharmacovigilance: establishing a conceptual and methodological framework | European Journal of Clinical Pharmacology | N/A (Netherlands) | 1-1 | T | 3 | P | - | - | 2 | O | - |

| Authors (year) | Title | Journal | Country | Field | Article type | Article aim | Theory Model Framework | KM strategy type | KM strategy aim | KM strategy target | Components mentionned | Components evaluated |
|---|---|---|---|---|---|---|---|---|---|---|---|---|
| Buckley et al. (2003) | Applying a 'stages of change' model to enhance a traditional evaluation of a research transfer course | Journal of Evaluation in Clinical Practice | Canada (Canada) | 1-3 | E | 2 | C | 1 | A, P | 1 | C,O | C,O |
| Colquhoun et al. (2014) | Towards a common terminology: a simplified framework of interventions to promote and integrate evidence into health practices, systems, and policies | Implementation Science | N/A (Canada) | 2 | T | 3 | P,D | - | - | 3 | C,P,O,I | - |
| Couineau & Forbes (2011) | Using Predictive Models of Behavior Change to Promote Evidence-Based Treatment for PTSD | Psychological Trauma: Theory, Research, Practice, and Policy | Australia (Australia) | 1-1 | E | 2 | P | 1,3 | P | 1 | O | O |
| Cullen et al. (2022) | Iowa Implementation for Sustainability Framework | Biomedcentral | USA (USA) | 2 | E | 1 | P | - | M | 1 | C,P,O | - |
| Currie et al. (2005) | A model of impacts of research partnerships in health and social services | Evaluation and Program Planning | N/A (Canada) | 1-2 | T | 3 | E | 2 | P, C | 2 | P,O,I | - |
| Dadich (2010) | From bench to bedside: Methods that help clinicians use evidence-based practice | Australian Psychologist | N/A (Australia) | 1-1 | S | 3 | P | 1,2,3,4,5,7 ,8 | M | 2 | O,I | - |
| de la Garza et al. (2023) | Using the project ECHO™ model to teach mental health topics in rural Guatemala: | International journal of social Psychiatry | Guatemala (Guatemala) | 1-3 | E | 2 | E | 1 | I,A | 1 | P,O | P,O |

| Authors (year) | Title | Journal | Country | Field | Article type | Article aim | Theory Model Framework | KM strategy type | KM strategy aim | KM strategy target | Components mentionned | Components evaluated |
|---|---|---|---|---|---|---|---|---|---|---|---|---|
| | An implementation science-guided evaluation | | | | | | | | | | | |
| Dobbins et al. (2002) | A Framework for the Dissemination and Utilization of Research for Health-Care Policy and Practice | The Online Journal of Knowledge Synthesis for Nursing | N/A (Canada) | 1-2 | T | 3 | P | - | - | 2 | C,P,O,I | - |
| Dufault (2004) | Testing a Collaborative Research Utilization Model to Translate Best Practices in Pain Management | Worldviews on Evidence-Based Nursing | N/A (USA) | 1-1 | T | 4 | P | 1 | P | 1 | P,O,I | - |
| Duhamel et al. (2015) | Integrating the Illness Beliefs Model in Clinical Practice: A Family Systems Nursing Knowledge Utilization Model | Journal of Family Nursing | Canada (Canada) | 1-1 | E | 2 | P | 1,4 | A, P | 1 | O,I | O,I |
| Dwan et al. (2015) | Measuring the success of facilitated engagement between knowledge producers and users: a validated scale | Evidence & Policy | Australia (Australia) | 1-2 | E | 2 | C | 1 | I | 1 | C,O | C,O |
| Edgar et al. (2006) | The Joint Venture Model of Knowledge Utilization: A Guide for Change in Nursing | Nursing Research | N/A (Canada) | 1-1 | T | 4 | D | - | - | 2 | C,O,I | - |
| Farkas et al. (2003) | Knowledge Dissemination and Utilization in Gerontology: An Organizing Framework | The Gerontologist | USA (USA) | 1-1 | T | 4 | P | - | M | 2 | - | - |

| Authors (year) | Title | Journal | Country | Field | Article type | Article aim | Theory Model Framework | KM strategy type | KM strategy aim | KM strategy target | Components mentionned | Components evaluated |
|---|---|---|---|---|---|---|---|---|---|---|---|---|
| Gagliardi et al. (2008) | Fostering knowledge exchange between researchers and decision-makers: Exploring the effectiveness of a mixed-methods approach | Health Policy | Canada (Canada) | 1-1 | E | 1, 2 | D | 2,3 | C | 1 | C,P,O | O |
| Gagliardi et al. (2011) | Protocol: developing a conceptual framework of patient mediated knowledge translation, systematic review using a realist approach | Implementation Science | N/A (Canada) | 1-1 | S | 3 | P | - | M | 3 | I | - |
| Gagliardi et al. (2012) | The guideline implementability research and application network (GIRAnet): an international collaborative to support knowledge exchange: study protocol | Implementation Science | N/A (Canada) | 1-1 | E | 1 | P | 2 | M | 3 | P,O,I | P,O,I |
| Gainforth et al. (2015) | Examining the Feasibility and Effectiveness of a Community-Based Organization Implementing an Event-Based Knowledge Mobilization Initiative to Promote Physical Activity Guidelines for People With Spinal Cord Injury Among Support Personnel | Health Promotion Practice | Canada (Canada) | 1-1 | E | 2 | E | 1,6 | I | 1 | P,O | P,O |

| Authors (year) | Title | Journal | Country | Field | Article type | Article aim | Theory Model Framework | KM strategy type | KM strategy aim | KM strategy target | Components mentionned | Components evaluated |
|---|---|---|---|---|---|---|---|---|---|---|---|---|
| Garad et al. (2018) | Evaluation of a Center of Research Excellence in Polycystic Ovary Syndrome as a Large-Scale Collaborative Research Translation Initiative, Including Evaluating Translation of Guideline Impact | Seminars in Reproductive Medicine | N/A (Australia) | 1-1 | T | 4 | L | 1,2,3 | P | 1 | P,O,I | - |
| Glasgow et al. (2019) | RE-AIM Planning and Evaluation Framework: Adapting to New Science and Practice With a 20-Year Review | Frontiers in Public Health | N/A (USA) | 2 | T | 3 | E | - | - | 3 | C,P,O,I | - |
| Gonzales et al. (2012) | A framework for training health professionals in implementation and dissemination science. | Academic medicine: journal of the Association of American Medical Colleges | USA (USA) | 1-3 | T | 4 | D | 1 | P | 2 | C,O,I | - |
| Graham et al. (2006) | Lost in Knowledge Translation: Time for a Map? | The Journal of Continuing Education in the Health Professions | N/A (Canada) | 2 | T | 3 | P | - | - | 3 | C,O,I | - |
| Grooten et al. (2020) | The transfer of knowledge on integrated care among five European regions: a qualitative multi-method study | BMC Health Services Research | Europe (Belgium) | 1-1 | E | 2 | P | 1,2,4 | A, P, C | 1 | - | - |

| Authors (year) | Title | Journal | Country | Field | Article type | Article aim | Theory Model Framework | KM strategy type | KM strategy aim | KM strategy target | Components mentionned | Components evaluated |
|---|---|---|---|---|---|---|---|---|---|---|---|---|
| Guo et al. (2011) | Application of a logic model to an evidence-based practice training program for speech-language pathologists and audiologists | Association of Schools of Allied Health Professions | USA (USA) | 1-1 | E | 1, 2 | L | 1,3,5 | I, A, P | 1 | P,O,I | P,O* |
| Haines et al. (2012) | Determinants of successful clinical networks: the conceptual framework and study protocol | Implementation Science | Australia (Australia) | 1-1 | E | 1 | L | 1,2,3,6,7 | P | 1 | C,O,I | C,O,I |
| Haynes et al. (2020) | Knowledge mobilisation in practice: an evaluation of the australian prevention partnership centre | Health Research Policy and Systems | Australia (Australia) | 1-2 | E | 2 | L | 1,2,4,5 | M | 1 | C,P,I | C,P,I |
| Hinchcliff et al. (2017) | The enhanced knowledge translation and exchange framework for road safety: a brief report on its development and potential impacts | BMJ- injury prevention | Europe (Australia) | 1-2 | E | 1 | D | 1 | D, P, C | 2 | P,O | - |
| Ho et al. (2004) | Technology-Enabled Knowledge Translation: Frameworks to Promote Research and Practice | Health Research Policy and Systems | N/A (Canada) | 1-1 | T | 3 | P | 1 | I | 2 | P,O,I | - |
| Imani-Nasab et al. (2017) | Validity and reliability of the Evidence Utilisation in Policymaking Measurement Tool (EUPMT) | Health Research Policy and Systems | Iran (Iran) | 1-2 | E | 1 | C | - | D | 2 | C,O | C,O |

| Authors (year) | Title | Journal | Country | Field | Article type | Article aim | Theory Model Framework | KM strategy type | KM strategy aim | KM strategy target | Components mentionned | Components evaluated |
|---|---|---|---|---|---|---|---|---|---|---|---|---|
| Jeffs et al. (2013) | Using theory and evidence to drive measurement of patient, nurse and organizational outcomes of professional nursing practice | International Journal of Nursing Practice | Canada (Canada) | 1-1 | T | 4 | E | 1,2,3,5 | P | 2 | I | I |
| Ko et al. (2019) | An application of the Science Impact Framework to the Cancer Prevention and Control Research Network from 2014-2018. | Preventive Medecine | USA (USA) | 1-2 | E | 2 | L | 1,2,3,4,6 | I,P | 1 | C,P,O,I | - |
| Kok & Schuit (2012) | Contribution mapping: a method for mapping the contribution of research to enhance its impact | Health Research Policy and Systems | N/A (Netherlands) | 1-1 | T | 3 | P | 6 | C | 2 | C,P,O | - |
| Kramer et al. (2013) | Did You Have an Impact? A Theory-Based Method for Planning and Evaluating Knowledge-Transfer and Exchange Activities in Occupational Health and Safety | International Journal of Occupational Safety and Ergonomics (JOSE) | Canada (Canada) | 1-1 | E | 1 | P | 3,4 | I, C | 1 | C,O | C,O |
| Kuchenmüler et al. (2022) | A comprehensive monitoring and evaluation framework for evidence to policy networks | Evaluation and program planning | Europe (Danemark) | 2 | E | 1 | L | 2 | I,C | 2 | C,O,I | - |

| Authors (year) | Title | Journal | Country | Field | Article type | Article aim | Theory Model Framework | KM strategy type | KM strategy aim | KM strategy target | Components mentionned | Components evaluated |
|---|---|---|---|---|---|---|---|---|---|---|---|---|
| Labbé et al. (2020) | Examining the Impact of Knowledge Mobilization strategies to inform urban stakeholders on accessibility: a mixed-methods study | International journal of environmental research and public health | Canada (Canada) | 1-2 | E | 2 | P | 6 | I, A | 1 | P,O | P,O |
| Langer & Weyrauch (2020) | Using evidence in Africa: a framework to assess what works, how and why (chap 3) IN Using Evidence in Policy and Practice (lessons from Africa) | Routledge (Taylor & Francis Group)<br>Titre livre : Using evidence in Policy and practice | African countries (South Africa) | 1-2 | T | 4 | P | - | M | 3 | O,I | - |
| Lavis et al. (2003) | Measuring the impact of health research | Journal of Health Services Research & Policy | N/A (Canada) | 1-2 | T | 4 | P,E | - | - | 3 | P,O,I | - |
| Leeman et al. (2012) | An Evaluation Framework for Obesity Prevention Policy Interventions | Preventing Chronic Disease<br>CDC- (Centres for disease control and prevention) | USA (USA) | 1-2 | T | 4 | L | 3 | M | 2 | P,O,I | - |
| Levin et al. (2011) | Fostering Evidence-Based Practice to Improve Nurse and Cost Outcomes in a Community Health Setting | Nursing administration Quarterly | USA (USA) | 1-1 | E | 2 | E | 1,4 | A | 1 | C,P,O,I | C,P,O |
| McCabe et al. (2015) | A model for collaborative working to facilitate knowledge mobilisation in public health | Evidence & Policy | UK (England) | 1-1 | E | 1, 2 | D | 2 | M | 1 | C,O | - |

| Authors (year) | Title | Journal | Country | Field | Article type | Article aim | Theory Model Framework | KM strategy type | KM strategy aim | KM strategy target | Components mentionned | Components evaluated |
|---|---|---|---|---|---|---|---|---|---|---|---|---|
| McDonald et al. (2010) | Building capacity for evidence generation, synthesis and implementation to improve the care of mothers and babies in South East Asia: methods and design of the SEA-ORCHID Project using a logical framework approach | BMC Medical Research Methodology | South East Asia (Australia) | 1-1 | T | 4 | P,E | 1,5 | P | 2 | C,P,O,I | - |
| Mfuso-Bengo et al. (2023) | Proposing the "Value- and Evidence-Based decision making and Practice" (VEDMAP) framework for Priority-Setting and knowledge translation in low and Middle-Income Countries: A novel framework for Decision-Making in Low-and middle income countries like Malawi. | Health Policy Open | Malawi (Malawi) | 1-2 | E | 1 | D | 2 | D | 1 | C,P | - |
| Moore et al. (2009) | Achieving Desired Results and Improved Outcomes: Integrating Planning and Assessment Throughout Learning Activities | Journal of continuing education in the health professions | N/A (USA) | 1-3 | T | 4 | P,E | 1 | P | 3 | C,O,I | - |

| Authors (year) | Title | Journal | Country | Field | Article type | Article aim | Theory Model Framework | KM strategy type | KM strategy aim | KM strategy target | Components mentionned | Components evaluated |
|---|---|---|---|---|---|---|---|---|---|---|---|---|
| Moreland-Russell et al. (2023) | A conceptual model for building program sustainability in public health settings: Learning from the implementation of the program sustainability action planning model and training curricula | Frontiers in health services | USA | 1-2 | E | 1-4 | L | 1,4 | P | 2 | C,O,I | O |
| Nadalin Penno et al. (2022) | Sustaining a nursing best practice guideline in an acute care setting over 10 years: A mixed methods case study | Frontiers in health services | Canada (Canada) | 1-1 | E | 2 | E | 1,3 | I,D | 1 | C,P | C,P |
| Paquette-Warren et al. (2016) | Case study of evaluations that go beyond clinical outcomes to assess quality improvement diabetes programmes using the Diabetes Evaluation Framework for Innovative National Evaluations (DEFINE) | Journal of Evaluation in Clinical Practice | Canada (Canada) | 1-1 | E | 1 | E | 1,2,3 | P | 1 | P,I | - |
| Paquette-Warren et al. (2017) | The Diabetes Evaluation Framework for Innovative National Evaluations (DEFINE): Construct and Content Validation Using a Modified Delphi Method | Canadian journal of Diabetes (2017) | Canada (Canada) | 1-1 | E | 1 | E | - | - | 2 | - | - |

| Authors (year) | Title | Journal | Country | Field | Article type | Article aim | Theory Model Framework | KM strategy type | KM strategy aim | KM strategy target | Components mentionned | Components evaluated |
|---|---|---|---|---|---|---|---|---|---|---|---|---|
| Pettman et al. (2016) | Evaluation of a knowledge translation and exchange platform to advance non-communicable disease prevention | Evidence & Policy | Australia (Australia) | 1-1 | E | 1 | L | 1,3,4 | I, A, P, C | 1 | C,P,O | C,P,O |
| Proctor et al. (2011) | Outcomes for Implementation Research: Conceptual Distinctions, Measurement Challenges, and Research Agenda | Adm Policy Ment Health | N/A (USA) | 2 | T | 3 | E | - | - | 3 | C,P,O,I | - |
| Reddy et al. (2015) | Evaluating impact of clinical guidelines using a realist evaluation framework | Journal of evaluation in clinical practice | Australia (Australia) | 1-1 | E | 2 | L | 1 | P | 1 | O | O |
| Richard et al. (2014) | Communities of Practice as a Professional and Organizational Development Strategy in Local Public Health Organizations in Quebec, Canada: An Evaluation Model | HEALTHCARE POLICY | Canada (Canada) | 1-1 | T | 4 | E | 1 | P, C | 2 | C,P,O,I | - |
| Rosella et al. (2018) | Evaluating the Process and Outcomes of a Knowledge Translation Approach to Supporting Use of the Diabetes Population Risk Tool (DPoRT) in Public Health Practice | Canadian Journal of Program Evaluation | Canada (Canada) | 1-1 | E | 1, 2 | P | 1,2,4 | P | 1 | C,P,O | C,P,O |

| Authors (year) | Title | Journal | Country | Field | Article type | Article aim | Theory Model Framework | KM strategy type | KM strategy aim | KM strategy target | Components mentionned | Components evaluated |
|---|---|---|---|---|---|---|---|---|---|---|---|---|
| Rycroft-Malone et al. (2013) | Collaborative action around implementation in Collaborations for Leadership in Applied Health Research and Care: towards a programme theory | Journal of Health Services Research & Policy | UK (Wales) | 1-1 | E | 2 | P | 2 | C | 1 | C,P,O | - |
| Sargeant et al. (2011) | CPD and KT: Models Used and Opportunities for Synergy | Journal of continuing education in the health professions | N/A (Canada) | 1-3 | T | 3 | P,E | - | - | 3 | O,I | - |
| Shelton et al. (2020) | An Extension of RE-AIM to Enhance Sustainability: Addressing Dynamic Context and Promoting Health Equity Over Time. | Frontiers in Public Health | USA (USA) | 1-2 | T | 4 | E | - | - | 2 | P,O | P,O |
| Sketris et al. (2020) | Building a framework for the evaluation of knowledge translation for the Canadian Network for Observational Drug Effect Studies | Pharmacoepidemiolo gy Drug Safety | Canada (Canada) | 1-1 | T | 3 | L | - | M | 2 | C,P,O | - |
| Skiner (2007) | Developing a tool to measure knowledge exchange outcomes | The Canadian Journal of Program Evaluation | N/A (Canada) | 1-1 | T | 3 | E | 1 | I | 3 | O,I | O,I |
| Smith et al. (2009) | The Kirkpatrick model: A useful tool for evaluating training outcomes | Journal of Intellectual and Developmental Disability | N/A (Australia) | 1-3 | E | 1, 2 | P | 1 | P | 1 | O,I | - |

| Authors (year) | Title | Journal | Country | Field | Article type | Article aim | Theory Model Framework | KM strategy type | KM strategy aim | KM strategy target | Components mentionned | Components evaluated |
|---|---|---|---|---|---|---|---|---|---|---|---|---|
| Stetler (2001) | Updating the Stetler Model of Research Utilization to Facilitate Evidence-Based Practice | NURSING OUTLOOK | N/A (USA) | 2 | T | 4 | P | - | - | 3 | C,P,O,I | - |
| Stetler et al. (2007) | Improving quality of care through routine, successful implementation of evidence-based practice at the bedside: an organizational case study protocol using the Pettigrew and Whipp model of strategic change | Implementation Science | USA (USA) | 1-1 | E | 1 | P | - | P | 1 | C,P,O,I | C,P |
| Stetler et al. (2011) | A Guide for applying a revised version of the PARIHS framework for implementation | Implementation Science | N/A (USA) | 2 | T | 3 | D | - | - | 3 | C,P,I | - |
| Straus et al. (2010) | Monitoring use of knowledge and evaluating outcomes | *CMAJ* (*Canadian Medical Association Journal*) | N/A (Canada) | 2 | T | 3 | P | - | - | 3 | O,I | - |
| Talbott et al (2023) | Evidence-Based Assessment in Special Education Research: Advancing the Use of Evidence in Assessment Tools and Empirical Processes | Exceptional children | USA (USA) | 3 | T | 4 | P | - | - | 3 | P,O,I | - |
| Thomson et al. (2019) | Programme theory development and formative evaluation of a | Health Research Policy & Systems | Canada (Canada) | 1-2 | E | 1, 2 | L | 1,2 | P, C | 1 | P,O | - |

| Authors (year) | Title | Journal | Country | Field | Article type | Article aim | Theory Model Framework | KM strategy type | KM strategy aim | KM strategy target | Components mentionned | Components evaluated |
|---|---|---|---|---|---|---|---|---|---|---|---|---|
| | provincial knowledge translation unit | | | | | | | | | | | |
| Tschida & Drahota (2023) | Fidelity to the ACT SMART Toolkit: an instrumental case study Of implementation strategy fidelity | Implementation Science Communication | USA (USA) | 1-1 | E | 1 | P | 1,4 | P | 1 | P | P |
| Van Eerd et al., (2021) | A research impact model for work and health | American journal of industrial medicine | Canada (Canada) | 1-1 | T | 4 | L | - | I | 2 | C,O,I | - |
| Varallyay et al. (2020) | Health system decision-makers at the helm of implementation research: development of a framework to evaluate the processes and effectiveness of embedded approaches | Health Research Policy & Systems | Latin America and the Caribbean (USA) | 1-2 | E | 1, 2 | E | 2,3 | D | 1 | C,P,O,I | - |
| Ward et al. (2012) | Exploring knowledge exchange: A useful framework for practice and policy | Social Science & Medicine | UK (england) | 1-1 | E | 1 | P | 4 | I, C | 1 | - | - |

| Authors (year) | Title | Journal | Country | Field | Article type | Article aim | Theory Model Framework | KM strategy type | KM strategy aim | KM strategy target | Components mentionned | Components evaluated |
|---|---|---|---|---|---|---|---|---|---|---|---|---|
| Ward (2017) | Why, whose, what and how? A framework for knowledge mobilisers | Evidence & Policy | N/A (England) | 2 | S | 3 | P | - | M | 3 | - | - |
| Wimpenny et al. (2008) | Tracing and Identifying the Impact of Evidence—Use of a Modified Pipeline Model | Worldviews on Evidence-Based Nursing | N/A (Scotland) | 2 | T | 3 | P | - | - | 2 | O,I | - |
| Ye et al. (2022) | Identifying Contextual Factors and Strategies for Practice Facilitation in Primary Care Quality Improvement Using an Informatics-Driven Model: Framework Development and Mixed Methods Case Study | JMIR Human Factors | USA (USA) | 1-1 | E | 1 | D | 1,4 | P | 1 | C,P | C,P |
| Yearwood (2018) | Applying a logical theory of change for strengthening research uptake in policy: a case study of the Evidence Informed Decision Making Network of the Caribbean. | Pan American Journal of Public Health | Caribbean (Trinidad and Tobago) | 1-2 | E | 1 | L | 1,2 | M | 1 | O,I | O* |
| Yip et al. (2021) | A Contextual Analysis and Logic Model for Integrated Care for Frail Older Adults Living at Home: The INSPIRE Project | International journal of integrated care | Europe (Suisse) | 1-1 | T | 4 | L | - | I | 1 | C,O,I | - |

| Authors (year) | Title | Journal | Country | Field | Article type | Article aim | Theory Model Framework | KM strategy type | KM strategy aim | KM strategy target | Components mentionned | Components evaluated |
|---|---|---|---|---|---|---|---|---|---|---|---|---|
| Yu et al. (2022) | Comprehensive Evaluation on Teachers' Knowledge Sharing Behavior Based on the Improved TOPSIS Method. | Computational Intelligence and Neuroscience | Chine (Chine) | 3 | E | 1 | E | - | - | 1 | O | O |
